# Non-Steroidal Anti-Inflammatory Drugs in Alzheimer's Disease and Parkinson's Disease: Reconsidering the Role of Neuroinflammation

**DOI:** 10.3390/ph3061812

**Published:** 2010-06-02

**Authors:** Amy H. Moore, Matthew J. Bigbee, Grace E. Boynton, Colin M. Wakeham, Hilary M. Rosenheim, Christopher J. Staral, James L. Morrissey, Amanda K. Hund

**Affiliations:** Department of Biology, Carleton College, one north college street, Northfield, MN 55057, USA

**Keywords:** Non-steroidal anti-inflammatory drugs, Alzheimer's disease, Parkinson's disease, cyclooxygenase, neuroinflammation

## Abstract

Alzheimer's disease (AD) and Parkinson's disease (PD) are the most common neurodegenerative diseases with age as the greatest risk factor. As the general population experiences extended life span, preparation for the prevention and treatment of these and other age-associated neurological diseases are warranted. Since epidemiological studies suggested that non-steroidal anti-inflammatory drug (NSAID) use decreased risk for AD and PD, increasing attention has been devoted to understanding the costs and benefits of the innate neuroinflammatory response to functional recovery following pathology onset. This review will provide a general overview on the role of neuroinflammation in these neurodegenerative diseases and an update on NSAID treatment in recent experimental animal models, epidemiological analyses, and clinical trials.

## 1. Introduction

According to the United States Centers for Disease Control and Prevention, the average American lifespan has increased from 73.7 years (1980) to 77.9 years (2007) [1]. Aging is associated with increased risk of neurological disease or disorder, including Alzheimer's disease (AD) and Parkinson's disease (PD). Collectively, these neurodegenerative diseases are estimated to impact over 10% of the US population >60 years of age, at an annual cost beyond $30 billion USD related to medical care, lost earnings, and compensation for lost earnings [2,3]. Of course, there exists no data or concrete price for the time, effort, and emotion provided by family members and caregivers. Therefore, identification of preventive and treatment strategies is of great interest to address disease-associated medical and mental health needs, while maintaining quality of life during extended life span. 

Although the etiology and behavioral symptoms differ among neurodegenerative diseases, neuroinflammation is a feature of both AD and PD. Neuroinflammation, as characterized by activation of glia (gliosis) and elevated presence of inflammatory molecules, is a common component of the normal aging brain, yet is exacerbated in AD and PD. Analogous to stimulated macrophages in peripheral immune responses, reactive microglia mediate central nervous system (CNS) immune responses through the phagocytosis of necrotic material and the release of pro-inflammatory signals to initiate "wound healing". In this respect, it is not surprising to observe inflammatory markers in conjunction with disease pathology and microglia containing fragments of cellular debris in regions of neurodegeneration. Although assumed to be a local tissue response to combat the condition-specific pathology, neuroinflammation independently appears to actively contribute to CNS pathophysiology. Consideration of age-associated reactive gliosis in light of epidemiological studies reporting reduced risk of AD in patients with chronic use of non-steroidal anti-inflammatory drugs (NSAIDs) [4] has spurred intense research to investigate the implications of neuroinflammation. Furthermore, the development of specific NSAIDs and the recognition of NSAID targets in the diseased brain have fueled a wide range of animal and clinical studies. This overview will provide an update on the understanding of neuroinflammation's role in selected neurodegenerative diseases by highlighting experimental and clinical results that illustrate the impact of NSAID administration on AD and PD progression. 

## 2. NSAID Mechanism of Action

Rubor (redness), calor (heat), tumour (swelling), and dolor (pain) were the cardinal signs of inflammation described by the Romans (30–40 B.C.) [5]. The use of medicines to alleviate peripheral inflammation is one of the oldest therapies on record, with chewing of willow leaves and bark prescribed by Hippocrates to reduce pain, swelling, and fever. Although the active ingredient in willow bark extract, salicin, was isolated in the early 19th century and mass distributed as acetylsalicylic acid under the brand name Aspirin^®^ (Bayer) by the end of the century, the mechanism by which this NSAID relieved inflammatory symptoms was unknown. In 1971, John Robert Vane determined that administration of aspirin decreased prostaglandin synthesis, thus implicating prostaglandins as crucial in the inflammatory pathway [6]. 

**Figure 1:**
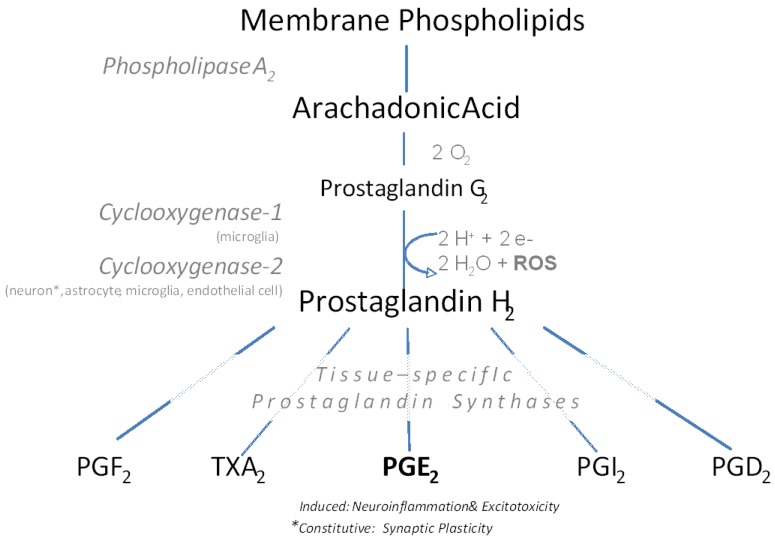
Prostaglandin synthesis with attention to cyclooxygnease expression in neural cells and function of PGE_2_ in brain. Modified from [8].

The synthesis of prostanoids (prostaglandins and thromboxanes), a group of potent lipid mediators acting in diverse physiological processes, is dependent on the enzymatic activity of cyclooxygenase (COX). The COX enzyme catalyzes two sequential reactions in different active sites [7]. In the first reaction, arachadonic acid (AA) forms extensive van der Waals interactions with cyclooxygenase and is sequestered from the solvent, converting AA into prostaglandin (PG) G_2_. The second peroxidase reaction converts PGG_2_ into PGH_2_. Action of tissue-specific isomerases/synthases is the final step of prostanoid production (Figure 1). Acting through G-coupled receptors, prostanoids influence constitutive functions in the periphery, including mucous production in the gastrointestinal (GI) tract, vasoregulation, and modulation of endocrine signaling. However, inflammation is primarily associated with a rise in tissue PGE_2_ levels, designating PGE_2_ as the pro-inflammatory PG. In the CNS, induction of PGE_2_ corresponds to disease pathology, traumatic injury, and inflammatory stimulus.

In 1991, molecular studies revealed that the COX enzyme exists in multiple isoforms, identified as COX-1 and COX-2 (for a review, see [9]). In addition, tissue-specific expression of COX-3, an additional isoform produced by a splice variant of COX-1, was described in 2002 [10] however it is not believed to contribute to PG production [11] and will not be considered further in this review. The amino acid sequences of COX-1 and COX-2 are more than 60% identical, governing similar overall structure. When considering genetic sequences, COX-2 has a unique promoter region that allows upregulation of expression by growth factors, tumor promoters, hormones, bacterial endotoxin, cytokines, anoxia, neurotoxins, electrical stimulation, and pro-inflammatory stimuli [12]. Therefore, it has been accepted that COX-1 is constitutively expressed whereas COX-2 is expressed as part of the inflammatory response in most tissues. However, these traditional roles of COX isoform expression are not observed in the brain as COX-2 mRNA and protein are detectable in neurons in absence of inflammatory stimulus [13,14,15] and COX-1 has been reported to contribute to elevations of PGE_2_ in experimental models of neuroinflammation [16,17].

**Figure 2:**
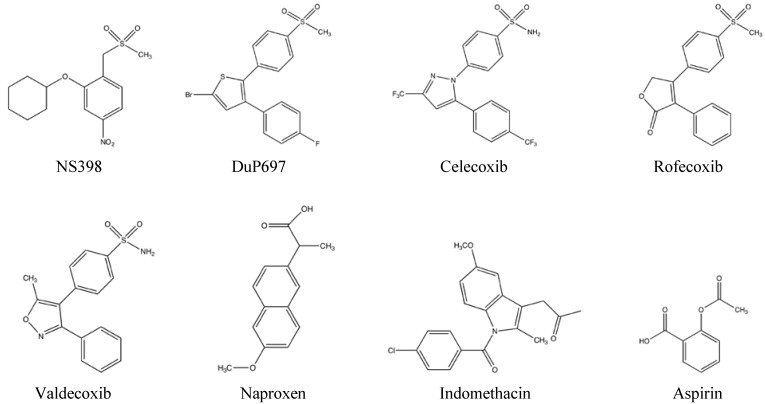
Chemical structures of common NSAIDs

Although having effective anti-pyretic, anti-inflammatory, anti-thrombotic and analgesic properties, chronic aspirin use was associated with GI discomfort, ulcers, and bleeding due to reduction of prostaglandins required for normal physiology. Pharmacological analysis determined that the acetyl group of aspirin irreversibly inhibits COX from binding AA. The introduction of ibuprofen, a reversible non-specific COX inhibitor, provided an alternative NSAID with reduced side effects. However, the discovery of COX-2 as the inducible and pro-inflammatory enzyme isoform fueled intense work to develop COX-2 selective inhibitors for maximum inflammation resolution with minimal gastric side effects. X-ray crystallography data has shown that the COX-2 isoform contains a small structural difference in the binding site of arachidonic acid; it contains an extended pocket in the binding site, which allows COX-2 selective inhibitors to be constructed with a proturbance that would fit into this pocket [18]. Many COX-2 selective inhibitors contain a 4-methylsulfonyl or sulfonamide substiuent on a cis-stilbene moiety that fits into this pocket [19] (Figure 2). Examples of drugs in this class include NS398, DuP697, celecoxib, rofecoxib, and valdecoxib with varying selectivies for COX-2 (Table 1). 

**Table 1:** COX-2 selectivity of common NSAIDs. A higher ratio value indicates electivity or COX-2. Table modified from [20,21].

NSAID	COX-1 / COX-2 IC_50_ Ratio
Aspirin	0.006
Indomethacin	0.017–0.45
Ibuprofen	0.067–1.50
Diclofenac	0.45–1.43
Naproxen	1.7
Meloxicam	3–77
Celecoxib	350
Rofecoxib	1000

## 3. Overview of Neuroinflammation

Although not exhibiting the same cardinal symptoms as peripheral inflammation, neuroinflammation is integral in the brain's response to cellular stress, injury, and pathology. A hallmark of neuroinflammation is activation of astrocytes and microglia, collectvely termed reactive gliosis. Astrocytes, the most abundant cells in the CNS, serve multiple constitutive functions such as contributing to the structure and preservation of the blood-brain barrier, buffering and maintaining homeostasis of the extracellular environment, generating energy substrates in conditions of functional demand, and contributing to synaptic stability. Microglia, the resident macrophages of the brain, maintain a low profile in basal conditions, existing as surveyors and scavengers by identifying and engulfing cellular debris. However, in the presence of an inflammatory stimulus, astrocytes and microglia demonstrate a reactive phenotype that is characterized by a more spherical cell soma, hypertrophy of nuclei, and elongation/extension of processes (for review, see [22]). 

In addition to morphological changes, reactive glia produce a variety of molecules that trigger and contribute to chronic inflammation. Termed "the cytokine cycle" [23], glial-derived pro-inflammatory cytokines, such as interleukin (IL)-1beta, IL-6, tumor necrosis factor(TNF)-alpha, and transforming growth factor (TGF)-beta, set into motion a spectrum of signaling events that continuously feedback and influence each other in neurological disease and disorder (for reviews, see [24,25]. One such pathway is induction of COX-2 in neuronal and non-neuronal cells with subsequent increase in pro-inflammatory PGE_2_ [26]. Receptors for PGE_2_ (EP1-4) are found on microglia, astrocytes, and select neuronal populations [26]. Under normal circumstances, PGE_2_ signaling modifies synaptic plasticity in neuronal populations [27,28,29,30] implicated in learning and memory (for review, see [8]). However, following an inflammatory stimulus, EP receptor activation on both microglia and neurons appears to contribute to pro-inflammatory progression and neurotoxicity [31,32]. 

It is critical to note that reactive glia can also create cellular damage by contributing to the formation of reactive oxygen species (ROS) (Figure 1). Pro-inflammatory cytokines induce nitric oxide synthase (iNOS) and COX-2 activity in astrocytes, leading to an increase in free radical formation and oxidative stress in several neurodegenerative states [33,34,35]. In addition to direct damage, ROS can dramatically influence cell signaling pathways, particularly those involved in cytokine and other inflammatory modulator production (*i.e.,* PGE_2_), and accelerate neuronal death.

Due to the consistent appearance of neuroinflammation in neurodegenerative disease, players in the neuroinflammatory cascade have become potential therapeutic targets. Since many NSAIDs cross the mammalian BBB rapidly [36], COX-mediated PG synthesis has been at the forefront of consideration for enhanced understanding of normal and diseased brain function. However, animal and clinical studies have revealed that neuroinflammation in neurodegenerative disease holds great complexity, with NSAID efficacy on pathology and behavior varying by disease, experimental model, time, and neural region. 

## 4. Alzheimer's Disease

### 4.1. Disease impact and pathology

Alzheimer's disease, the most common cause of dementia [37], is a major public health concern. In 2006, a report by the World Health Organization estimated that worldwide over 20 million people suffer from AD and other dementias [38], including an estimated 5.3 million Americans [2], 95% of whom are over 65 years of age. In the United States, the average annual cost per patient equals approximately US $24,500, and total expenditures on care for AD reached US $84 billion in 2005 [39]. Patients with AD display progressive memory loss and behavioral changes that eventually compromise daily life. The cause of AD is still unknown. A genetic component is linked to less than 1% of all cases. However, age is the greatest risk factor and it is assumed that multiple physiological issues that occur during aging contribute to AD [2].

The pathology that accompanies the cognitive and behavioral symptoms of AD was first described by Dr. Alois Alzheimer in 1906. Upon post-mortem analysis of a brain from a patient with documented memory impairment, Alzheimer observed gray matter loss with noticeable plaques (misshapen protein aggregates) in remaining tissue. Since these gross observations, AD pathology has been further studied and is now recognized by the presence of cerebrocortical atrophy in the temporal lobe and surrounding regions that is associated with extracelullar beta-amyloid (Aβ) fibrils and abnormal levels of intracellular tau protein filaments that form senile plaques and neurofibrillary tangles, respectively [40]. The stereotypical "plaques and tangles" compromise intracellular transport within neurons and synaptic integrity between neurons, leading to impairment of neuronal function and associated cognitive impairments. These cellular/pathological characteristics have been confirmed using immunochemical techniques on post-mortem brain as well as with non-invasive imaging in patients displaying mild cognitive impairment or with familial predisposition for AD [41,42,43].

### 4.2. Animal models investigating neuroinflammation in AD

When epidemiological studies reported that chronic NSAID use reduces risk for AD [4], increasing attention was focused on markers of neuroinflammation in the AD brain. Numerous rodent models have been utilized to better understand the relationship between inflammatory mediators and pathology observed in post-mortem brain from dementia and AD patients [44,45], including direct administration of Aβ, infusion of pro-inflammatory agents to brain, and genetically modified rodent strains that over- or under-express the pathological markers of AD and their precursors [46,47]. The majority of these models shows reactive gliosis and altered brain expression of COX-2 that accompanies pathology and behavioral dysfunction. Considerable evidence suggests that the increase in COX-2 in such models is due to both direct and indirect activation of microglia by Aβ (reviewed in [48]). In addition to microgliosis, elevated COX-1 and COX-2 protein levels are observed in post-mortem AD brain compared to age-matched, non-demented controls [49,50,51]. Specifically, neuronal COX-2 expression in the hippocampus directly correlates with the severity of the dementia and COX-2 immunoreactivity in the CA1 correlates strongly with AD plaque and neurofibrillary tangle density [49]. 

As age is a risk factor for AD and an increase in glial activation has been observed in aged brain [45,52,53,54,55,56], studies have attempted to ascertain the causal relationship between neuroinflammatory markers and pathological markers for AD. Rodent models of chronic neuroinflammation have demonstrated induction of AD-like molecular and cellular changes [57,58] and spatial memory deficits [16,57,59]. Furthermore, glial activation can be observed prior to the detection of tau-related neurofibrillary tangles [60] and induction of neuroinflammation by both pro-inflammatory stimulus lipolysaccharide (LPS) and IL-1 increase the rate of tau phosphorylation, an intermediary step in the development of tangles [61,62]. 

Considering that COX-2 expression increases following microglial activation, it is of interest that transgenic mice over-expressing COX-2 developed an age-dependent deficit in spatial memory at 12 months and 20 months of age that was associated with remarkable neuronal apoptosis and astrocyctic activation [63]—results that suggest an independent contribution of COX-2 activity in neurodegeneration that may impact progression of AD pathology. Deletion or antagonists of the PGE_2_ receptors EP2 and EP4, both in cultured cells expressing mutant amyloid precursor protein (APP) [64,65] and *in vivo* [64], resulted in a significant reduction of beta-amyloid plaques, further confirming that Aβ and PGE_2_ synthesis are part of a positive feedback loop contributing to AD pathology.

To test this hypothesis, studies using AD animal models have incorporated NSAID administration (reviewed in [66]), hypothesizing that inhibition of COX activity will reduce disease-associated pathology. For example, experiments have demonstrated that oral administration of ibuprofen, a non-specific COX inhibitor, at onset of amyloid plaque formation decreased glial activation and plaque density in transgenic mice over-expressing APP, a crucial determinant of Aβ fibrils [67,68]. Indomethacin treatment attenuated microglial activation, restored disturbance in hippocampal long-term potentiation, and prevented working memory deficits associated with Aβ injections into the dentate gyrus of rats [69]. Demonstrating the synergistic effects of inflammation and plaques, intracerebroventricular administration of Aβ induced increases in COX-2 levels and memory impairment that were magnified upon coincident intraperitoneal administration of LPS. These compounded alterations were attenuated by pre-treatment with NS398, a COX-2-selective inhibitor [70], indicating that elevated COX-2 levels may be an intermediate between the pathological markers of AD and the common cognitive and behavioral symptoms. 

### 4.3. Clinical trials of NSAIDs in AD treatment and prevention

Both epidemiological and animal model research demonstrating benefits of NSAID treatment in AD provided justification for clinical trials in assessing the efficacy of COX inhibitors (specific and non-specific) in the treatment or prevention of AD (for a review, see [12]). In particular, the development of COX-2 inhibitors (coxibs) and the presence of elevated COX-2 in both post-mortem brain and AD animal models positioned this class of NSAIDs as promising therapy with minimal GI side effects. Despite this initial evidence of indomethacin having beneficial effects in slowing cognitive decline in patients with mild to moderate AD [71], large-scale clinical trials assessing cognitive outcomes following NSAID administration have been disappointing (Table 2). Double-blind, randomized, placebo-controlled trials using nonselective NSAIDs [72,73,74,75,76] and COX-2 specific inhibitors [72,77] have shown no significant effect on cognitive performance in AD patients. Furthermore, four years of rofecoxib (COX-2-specific inhibitor) treatment in patients demonstrating mild cognitive impairment did not delay the onset of AD [78]. These recent studies from larger populations confirm previous studies incorporating COX-2 inhibitors in smaller subject groups or for shorter duration (reviewed in [79,80]), suggesting that NSAID treatment is ineffectual once memory decline and associated pathology have already developed.

To determine if COX inhibition was effective as a prevention strategy, the Alzheimer's Disease Anti-inflammatory Prevention Trial (ADAPT) was designed as the first primary prevention trial to assess the association between NSAID and AD incidence, enrolling more than 2,500 cognitively normal elderly patients seventy years or older with one first-degree relative exhibiting dementia. Subjects were randomly administered naproxen sodium (non-specific COX-inhibitor), celecoxib (COX-2-specific inhibitor) or a placebo. Cognitive assessment during 1–3.5 years of treatment did not find a protective impact of celecoxib with respect to AD incidence nor cognitive performance [81]. In fact, the study indicated a cognitive deficit associated with naproxen treatment. Needless to say, these results were disappointing.

### 4.4. Consideration of clinical trials to determine future investigation of NSAIDs in AD

The recent clinical trials have placed doubt on the driving role of inflammatory processes (particularly those that are mediated by COX-2) in AD progression. However, the interpretation of the non-significant results have inspired further discussion and alternative perspectives on the relationship of NSAIDs and AD with respect to onset and duration of NSAID use, roles of COX-1 and COX-2 in neural function, and non-COX targets of specific NSAIDs.

It is critical to recognize that the ADAPT trial was prematurely halted due to increased risk for cardiovascular events (e.g. stroke, myocardial infarction) in unrelated clinical trials [82]. Therefore, the disappointing results may represent the truncated treatment duration relative to the prolonged NSAID use reported in the epidemiological studies. To revisit the basic tenet that microglial activation is a beneficial process following CNS insult, the ineffectiveness of NSAID treatment (COX-selective and non-selective) on AD progression may indicate that neuroinflammatory processes are present to combat AD-related cellular events. For example, sustained hippocampal microglial activation associated with IL-1β transgene expression in APPswe/PS1dE9 transgenic mice led to a significant decrease in soluble and insoluble Aβ plaque levels [83], demonstrating a possible adaptive and protective role of neuroinflammation in AD when initiated in the presence of developing AD pathology. Similarly, induction of systemic inflammation via LPS administration prior to Aβ intracerebroventricular infusion protected mice from COX-2 elevation and memory deficit [70]. Thus, the use of NSAIDs once patients exhibit AD-related symptoms or in the asymptomatic aged population where Aβ plaque formation has been initiated may reduce the beneficial effect of neuroinflammation on plaque degradation and removal. Even though subject numbers were small enough to warrant caution, Small and colleagues have reported that 12 months of celecoxib treatment attenuated age-related memory decline in patients (>65 years of age) who were not clinically impaired at the start of the study [84], suggesting that the potential window for NSAID efficacy is during normal aging prior to disease onset. The use of non-invasive imaging to detect amyloid burden in normal individuals may be crucial in identifying appropriateness for inclusion in future NSAID prevention trials [85].

**Table 2:** Overview of clinical trials (double-blind, placebo-controlled) that investigated NSAID treatment on cognitive measures related to Alzheimer's disease and dementia. Modified from [66].

Author	Year	Ref.	Patient description	Total number of patients recruited	Total number of patients included in analysis	NSAID	NSAID duration (months)	NSAID dose (mg/day)	Effect on Cognitive Outcome Measures
Rogers *et al.*	1993	[65]	Mild to moderate AD	44	28	Indomethacin	6	100–150	Significant improvement
De Jong *et al.*	2008	[67]	Mild to moderate AD	51	38	Indomethacin	12	100	Not significant
Pasqualetti er al.	2009	[68]	Mild to moderate AD	132	97	Ibuprofen	12	800	Not significant
Aisen	2002	[71]	Mild to moderate AD	40		Nimesulide	3	200	Not significant
Soininen	2007	[70]	Mild to moderate AD	425	328	Celecoxib	12	400	Not significant
Aisen *et al.*	2003	[66]	Mild to moderate AD	351	351				
						Rofecoxib	12	25	Not significant
						Naproxen	12	440	Not significant
Reines	2004	[69]		692	481	Rofecoxib	12	25	Not significant
Martin	2008	[76]	Normal cognition >70 years; one relative with dementia	2528	2117				
			
						Celecoxib	24	400	Not significant
									Not significant;
						Naproxen	24	400	Trend towards impairment
Thal	2005	[72]	MCI	1457	1457	Rofecoxib	48	25	Significant impairment
Small	2008	[77]	age-associated memory decline	88	40	Cele	18	200 or 400	Significant improvement associated with increased glucose metabolism in prefrontal cortex

The emphasis of COX-2-dependent neuroinflammation in AD has been assumed based on the constitutive and induced expression profiles of COX-1 and COX-2, respectively, in peripheral tissue. However, COX-2 is abundant in post-synaptic dendrites of forebrain neurons [13,14,15] in normal brain with COX-2-dependent PGE_2_ participating in long-term potentiation (LTP) and long-term depression of the hippocampus [28,30,86], a region critical for learning and memory. Observation that COX-2 inhibition leads to cognitive deficits in normal young rodents [87,88,89,90,91] implies that constitutive COX-2 activity is necessary for normal neural function. Thus, selective inhibition of pathology-induced COX-2 in neurons and astrocytes may also compromise activity necessary for normal cognitive function, leading to inconclusive behavioral assessment in studies of dementia.

In contrast, COX-1 is expressed at low levels in resting microglia and induced during inflammatory conditions, lending towards its consideration as a substantial contributor to neuroinflammatory conditions (reviewed in [92]). The relative success of clinical trials using the COX-1-preferential inhibitor indomethacin [71], recent epidemiological studies showing protective effects of aspirin and ibuprofen on cognitive decline [93,94,95], laboratory studies with AD-related transgenic mice treated with ibuprofen or minocycline (tetracycline antibiotic shown to inhibit microglia) [96,97], and evidence that COX-1 is specifically induced during experimental chronic neuroinflammation [16], may indicate the benefit of directly attenuating COX-1 activity and microglial activation. Yet, possible thresholds should be taken into account as heavy NSAID use may increase risk for dementia and AD [98]. Future pre-clinical studies will benefit from re-visiting the potential value of non-selective NSAID treatment and dose, with co-administration of proton pump inhibitors to block NSAID-related gastric ulcer disease [73,99], to characterize the physiological landscape that precedes and contributes to AD risk and pathology onset. 

With returned consideration of COX-1-mediated mechanisms in AD pathology, clearer understanding of the direct mechanisms of non-specific NSAIDs on pathophysiology will be necessary before additional clinical trials are designed. In addition to reduction of COX activity and associated production of free oxygen radicals and prostaglandins, a limited number of non-selective NSAIDs have been shown to impact other targets, such as γ secretase (enzyme responsbile for cleaving APP to Aβ), the transcription factors peroxisome proliferator-activator receptor(PPAR)-γ and nuclear factor (NF)-κB (for review, see [80,100]). As these factors contribute to numerous signaling pathways in different cell populations that relate to AD, identification of COX-independent NSAID actions may explain the apparent discrepancy between epidemiological studies that suggest the neuroprotection of non-specific COX inhibitors (ibuprofen, aspirin, indomethacin) and clinical trials that demonstrate little benefit of preferential COX-2-inhibitor treatment (refer to Table 2) in AD. For example, the ADAPT study authors had originally intended to use ibuprofen as the non-specific NSAID yet altered the treatment to naproxen due to manufacturer availability with "no reason *a priori* that naproxen should not be substituted for ibuprofen" [101]. However, since the onset of the study, ibuprofen and select other non-specific NSAIDs have been shown to directly interact with Aβ fibrils and reduce plaque burden via modification of APP processing to a greater degree than naproxen [68,102,103,104,105,106], implying potentially different outcomes between drugs. Yet no enhancement in neuroprotection of these Aβ-lowering NSAIDs in delaying cognitive dysfunction in patients with mild AD was determined in a cohort study [93] nor a recent clinical trial with 18 months of treatment [107]. Future research investigating chronic administration of this particular class of NSAIDs as preventative agents is warranted to understand the complex spectrum of NSAID action that may lead to reduction of AD pathology and symptoms.

A final consideration in targeting future investigation is to recognize pre-existing conditions and genetic predispositions in subject populations. For example, in addition to timeframe of NSAID exposure, it would be valuable to examine the potential impact of existing peripheral inflammatory conditions that prompted NSAID use in the original AD epidemiological cohorts examined relative to the strict selection criteria required for inclusion into the ADAPT trial [108]. Furthermore, NSAID therapy may have an impact on AD progression in a subset of at-risk population. Recently, studies have analyzed that a protective effect of NSAIDs was attributed to those subjects having the apolipoprotein E epsilon4 allele (APOE 4) [74,109,110]. APOE 4 is a genetic risk factor for AD with carriers of two or more alleles having an increased risk of developing AD [111]. As APOE encourages proteolysis of Aβ, future clinical studies may benefit from inclusion of APOE 4 carriers to determine the interaction between genetic predisposition and NSAID mechanism of action in AD diagnosis.

## 5. Parkinson's Disease

### 5.1. Disease impact and pathology

Described as "shaking palsy" by Galen in 175 A.D, Parkinson's disease (PD) became acknowledged as a medical condition following publication of "An Essay on the Shaking Palsy" by Dr. James Parkinson in 1817. Initial symptoms of PD are characterized by development of motor deficits, such as inability to initiate and sustain movement (bradykinesia), unintentional tremor at rest, shuffling gait, and postural difficulty. Although not usually included in PD symptoms, cognitive deficits often develop in late stage PD [112]. The cause of PD is unknown; although current hypotheses of cause range from genetic mutations, environmental toxins, oxidative stress and history of head trauma (for review, see [3]). Age is the greatest risk factor for PD with 1–2% of those over 65 years of age in the US are currently affected as incidence increases with age. Although the disease itself is not fatal, gradual motor deterioration decreases quality of life and increases risk of mortality [113]. 

A defining pathological feature of Parkinson's disease is the accumulation of the protein α-synuclein into filamentous "Lewy bodies" within neurons and glial cells [114]. Lewy bodies are abnormal cytosolic protein aggregates that disrupt brain functioning. The aggregation of α-synuclein, a highly conserved protein in humans, is considered to be a critical step in the development and pathogenesis of Lewy body diseases. It is thought that the accumulation of α-synuclein occurs in the early stages of Parkinson's disease [115]. A second defining pathological feature of Parkinson's disease, and one assumed to occur after Lewy body formation, is the loss of pigmented dopaminergic (DA) neurons of the substantia nigra, leading to a visually striking and uncharacteristically pale substantia nigra [116]. DA neurons contribute to the synaptic circuitry of the basal ganglia, a functional set of nuclei that influence motor control. PD-typical motor dysfunction typical does not appear until ~80% depletion of DA cells, indicating a substantial duration of pathophysiology that precedes behavioral symptoms. From the clinician's view, Parkinson's disease ˝ ... is so slowly progressive. If you could cut the rate of progression by 20 or 30%, you would add years to people's useful lifetime ... we just need to slow it down a little bit and we will buy a lot of important time˝ [117].

### 5.2. Animal models investigating neuroinflammation in AD

Since reactive microglia were first observed in the vicinity of degenerating dopaminergic substantia nigral regions of post-mortem brains from Parkinson's disease patients [118] and illicit drug users injected with 1-methyl-4-phenyl-1,2,3,6-tetrahydropyridine (MPTP) [119], the role of neuro-inflammation in dopaminergic cell death has been intensely studied (for reviews, see [120,121]). These microglia are morphologically transformed to an activated state, moving from a branched resting state to an activated amoeboid conformation and producing proinflammatory molecules (nitric oxide, TNF-α, IL-1β, IL-6, COX-2), which bind to receptors on dopaminergic neurons in the substantia nigra [122,123,124,125,126]. Interestingly, non-invasive molecular imaging has used a microglial-specific radiolabeled ligand to detect reactive gliosis in numerous brain regions of Parkinson's disease patients relative to age-matched healthy control subjects [127,128]. As the degree of *in vivo* inflammation did not correlate with indices of dopaminergic uptake or movement disorder, these findings suggest that microglial activation is present at early stages of the disease and may contribute to disease progression.

Microglial activation is a common pathological feature in animal models where Parkinson's disease-related pathology has been induced through intra-nigral injections of toxins, specifically endotoxin LPS, and neurotoxins MPTP, 1-methyl-4-phenylpyridinium (MPP+), and 6-hydroxydopamine (6-OHDA). To assess regional response to inflammatory stimulus, Kim *et al.* [129] injected LPS into various brain structures and found enhanced microglial activation and subsequent inflammation-mediated dopaminergic neural degeneration in the substantia nigra. Furthermore, there was a positive correlation between microglial density and neuronal susceptibility to LPS-induced toxicity. Similar outcomes have been observed in co-administration wiht MPTP, and 6-OHDA, leading to decreased tyrosine hydroxylase (TH), a marker for intact DA neurons, and increased relative COX-2 protein expression [130]. These results suggest that the increased vulnerability of mesencephalic neurons to inflammation-mediated neurodegeneration is, in part, due to the abundance of microglia in that brain region and region-specific microglial activation that may occur during normal aging [131].

The intracellular signaling cascades triggered by neurotoxin administration and microglial-derived proinflammatory cytokines can lead to the expression of COX-2 and production of pro-inflammatory PGE_2_ [132]. It is important to note that COX-1 activity promotes the majority of PGE_2_ synthesis pre- and post-MPTP injection without a change in protein expression [133], suggesting this isoform's contribution to the neuroinflammatory process specific to PD. However, COX-2 activity is also critical in PD-related neurotoxicity as COX-2-null transgenic mice show spared nigrostriatal cell loss following MPTP administration [134]. The mechanism by which increased PGE_2_ synthesis mediates DA neurodegeneration has yet to be elucidated as activation of PGE_2_ receptors subtypes have contrasting effects depending on cell type and experimental model [135]. The PGE_2_ receptors EP1 and EP2 have been detected in substantia nigra of rodent and human brains [136,137]. *In vitro,* activation of EP1 receptors on dopaminergic neurons isolated from embryonic rat midbrain increases susceptibility to 6-OHDA lesion [136]. Complementary, EP2 receptor activation appears crucial for microglial-mediated neurodegeneration [138,139]. Thus, inhibition of PGE_2_ production via NSAID administration may appear as beneficial. However, EP2 receptor agonists invoked neuroprotection in cultured dopaminergic neurons [137], suggesting differential outcomes of PGE_2_ signaling on dopaminergic neurons and necessity to investigate the dominant signaling pathway during PD-associated neuroinflammation. 

Independent of prostaglandin synthesis, COX-2 may contribute to DA neuronal loss through production of reactive oxygen species (ROS). Teismann and colleagues demonstrated that rofecoxib-treated mice demonstrated resistance to MPTP that was associated with decreased content of 5-cysteinyl-dopamine, a product of COX-oxidation of dopamine, without a decrease in microglial activation [133]. The oxidative stress hypothesis of PD is also supported by the observation that activated microglia produce ROS and reactive nitrogen species, as well as produce cytokines that induce neuronal nitric oxide synthase (for review, see [140]). Both dopamine synthesis and degradation entail the formation of reactive species, thus the presence of additional oxidative stress from microglial activation may increase vulnerability of nigrostriatal dopaminergic neurons to oxidative damage (for a review, see [141]). 

Animal models generally support the notion that NSAIDs protect against the dopaminergic neural degeneration that is induced by certain neurotoxins (for review, see [142]). However, the NSAID-associated neuroprotection appears to be dependent on duration, dose, and onset relative to neurotoxin exposure, as well as the specific NSAID utilized. In particular, aspirin (acetylsalicylic acid or its metabolite salicylic acid) has been extensively investigated and has repeatedly conferred protection from the neuronal damage induced by MPTP and 6-OHDA via reduction of ROS [143,144,145,146,147]. Although evidence points to scavenging of ROS scavenger and protection of mitochondrial function, the exact mechanism of aspirin's neuroprotective effect remains to be determined. Inconsistent results have been reported for indomethacin [143,148]. Interestingly, few studies have investigated the effectiveness of ibuprofen in animal models of PD, yet protection against MPP+ and 6-OHDA lesions was observed with high drug dose [135]. Although COX-2 expression and activity is increased following DA-targeted neurotoxin, COX-2 inhibitors have varied outcomes with NS398, meloxicam, celecoxib, and rofecoxib showing benefit in preserving cell viability and motor ability [133,147,149,150,151,152,153] or no effect [143,144,146]. Interestingly, COX-2 inhibitors were neuroprotective in the MPTP experimental model only when given prior to toxin administration, highlighting the importance of the early response in progression to cell death and the distinction of prevention *versus* treatment when considering NSAID strategy. 

### 5.3. Epidemiological observation of NSAIDs in PD incidence

To our knowledge, no clinical trials have been designed to test the feasibility of NSAIDs as preventative or treatment therapy. However, the early identification of activated microglia in post-mortem PD brain [118] in conjunction with pre-clinical studies supporting neuroinflammation's early role in dopaminergic neurodegeneration has stimulated numerous epidemiological studies that have evaluated the therapeutic utility of NSAIDs in PD. Chen and colleagues [154] assessed the PD risk associated with aspirin and non-aspirin (ibuprofen, indomethacin, naproxen, diflunisal) NSAID use in ~140,000 people over a period of 14–18 years and found that participants regularly using nonaspirin NSAIDs at the beginning of the study had a 0.55 relative risk of PD diagnosis in comparison to non-users. Additional investigation in a separate co-hort of comparable size yielded similar results with self-reported ibuprofen use associated with a 35% risk reduction [155]. Unfortunately, more recent observational analyses in smaller case-controlled studies have been inconsistent with some studies suggesting NSAID neuroprotection [156,157] while others reporting no significant impact [158,159,160] and even increased risk [161,162] associated with NSAID use and PD diagnoses. In fact, a meta-analysis of 11 different observational studies determined that NSAIDs (aspirin and non-aspirin) as a class does not seem especially effective at lowering the risk of developing Parkinson's disease. Yet, a separate meta-analysis of seven studies that assessed the impact of >1 year exposure to aspirin and non-aspirin on diagnosis of incident PD has reported a 15% reduction in risk of PD with non-aspirin use [163]. 

Specifically, ibuprofen as an independent NSAID may have a slight neuroprotective effect [164]. Further evidence for ibuprofen in delaying PD onset is expected by recent report that calculated a 40% reduction in PD risk in regular users of ibuprofen [165]. Investigation of ibuprofen mechanism(s) of action on PD-related pathophysiology and how they differ from other NSAIDs will be critical in designing future animal and clinical studies. For example, ibuprofen was the most effective non-aspirin NSAID in inhibiting α-synuclein fibrillization while also destabilizing existing α-synuclein fibrils *in vitro* [166]. If its anti-fibrillization effect was conserved *in vivo* as suggested by a recent experimental model in rat [167], it is possible that, independent or complementary to mediating glial activity and COX induction, ibuprofen could prevent α-synuclein aggregation and destabilize existing aggregates, thereby slowing disease progression or delaying disease onset.

### 5.4. Consideration of epidemiology to determine future investigation of NSAIDs in PD

Data from epidemiological and animal investigation collectively indicate that non-specific NSAID exposure prior to diagnosis or protein aggregation may impact progression to neurodegeneration. Pre-clinical studies must continue to investigate the mechanisms by which this mediation occurs with specific attention devoted to identifying time- and NSAID-specific relationships between drug and oxidative stress, reactive gliosis, and α-synuclein aggregation. The epidemiological studies emphasize chronic NSAID exposure for reduced risk of PD, a dose duration that does not seem apparent in animal studies. Similar to AD, age is the greatest risk factor for PD. However, few animal models incorporate the confounding factor of age. Since increased glial reactivity is associated with the aging brain and advanced age has been shown to exacerbate MPTP neurotoxicity in mouse and non-human primate [168,169], utilization of aged animals may be more realistic in assessing the effectiveness of NSAID therapy in PD.

To our knowledge, there are no clinical studies being proposed that evaluate NSAID efficacy in PD. Ideally, these proposals would be large-scale clinical trials that administer NSAIDs for several years and follow subjects into advanced age (with increased disease risk), applying uniform dose administration, motor outcome measures, and data collection to assess the potential of NSAIDs (specifically ibuprofen) as preventative agents of PD. Given the negative results in the AD clinical trials [72,78,81] and the halt of coxib use due to potential cardiovascular (CV) risk [82,170] it is unlikely that clinical trials involving COX-2 inhibitors in PD patients are being considered. Yet, it should be noted that a subsequent large scale meta-analysis of 39 studies involving more than 41,000 patients treated with celecoxib or placebo has suggested that there is no increase in CV disease risk associated with NSAID use [170]. The Prospective Randomized Evaluation of Celecoxib Integrated Safety *vs.* Ibuprofen or Naproxen (PRECISION) trial, hopes to resolve some of these issues by designing a large-scale clinical study that will determine whether any CV risks in patients with rheumatoid arthritis or osteoarthritis outweigh the potential anti-inflammatory benefits of NSAIDs [171]. As experimental studies in PD animal models suggest that COX-2-inhibitor treatment prior to MPTP exposure reduces DA cell loss [133,147,149,150,151,152,153], data from the PRECISION trial may be useful in considering general NSAID and coxib administration as a preventative therapy for PD.

## 6. NSAIDs in Other Neurological Disorders

In addition to AD and PD, considerable evidence exists that neuroinflammation and elevation of COX occur in acute neurological injuries, such as ischemic stroke and traumatic brain injury. Although not considered chronic degenerative diseases, recognizing the role of neuroinflammation in these neurological insults may provide insight on neural recovery that can be applied to other disease models.

Stroke is the third highest cause of death in the US, with American Stroke Association reporting that ischemic stroke (occlusion of an artery) accounts for 87% of all cases. The disruption of blood flow leads to excitotoxicty, upregulation of inflammatory cytokines, glial activation, and promotion peripheral immune cell entry into damaged neural tissue [172]. Studies have shown NSAID pre-administration reduces acute neuroinflammation and tissue damage (cell death, edema, lesion volume, and oxidative damage) in response to arterial occlusion, as a model of brain ischemia in a time- and COX isoform-specific manner [173,174,175,176,177,178]. Interestingly, COX-2 inhibition had little effect when ischemic insult occurred during chronic neuroinflammatory state due to COX-2 overexpression [175], suggesting that NSAID benefit after ischemic stroke may be limited in the aged population. There exist few case reports or medical research studies to determine if the experimental evidence is representative of the clinical situation.

Traumatic Brain Injury (TBI) is loosely defined as non-degenerative and non-congenital injury to the brain from an external force. Such trauma can lead to temporary or permanent cognitive, physical and psychosocial impairment associated with brain atrophy, substantial neuronal death, axon degeneration and chronic neuroinflammation [179,180,181,182]. Although a high percentage of specialists and family practitioners prescribe NSAIDs to TBI patients [183], studies in transgenic mice lacking either COX-1 or COX-2 have been unclear on the role of the enzyme as an appropriate therapeutic target [184,185]. Furthermore, NSAID application in experimental TBI has led to mixed results, with COX-2 inhibition improving [186], exacerbating [187], not affecting [188], or having within-study mixed response [189] on TBI-induced outcomes in rats. Ibuprofen treatment following TBI significantly worsened cognitive outcome of rats [190]. These discrepancies among studies reinforce the importance of timing of NSAID onset as well as the contributions of COX-1 and COX-2 in the neural response to traumautic insult—both beneficial and deleterious- as well as in normal physiologyof cognition.

## 7. Is There a Future for NSAIDs in Neurodegenerative Disease?

While the initial promise of NSAID administration as a treatment or prevention strategy for neurodegenerative disease has not yet been realized, continual evidence from epidemiological, clinical, animal model and *in vitro* studies affirms that neuroinflammation is intimately involved in AD and PD. However, this involvement appears to have both costs and benefits. Furthermore, the complicated relationship between NSAID use and onset of symptoms and pathology include consideration of pharmaceutical actions that are independent of inflammatory signaling.

Collective consideration of the negative results of COX-2 specific inhibition in AD prevention trials and the observed neuroprotection of the non-COX-specific inhibitor ibuprofen in PD diagnosis forces us to re-examine the time-dependent functions of both COX-1 and COX-2 in pathology-related brain inflammation and spared neural function, overshadowing these isoforms' traditional roles in peripheral inflammation. Choi, Aid and Bosetti [92] have proposed consideration of whether microglial activation is the primary or secondary response in determining appropriate therapeutic targets as each response is mediated by different COX isoforms. If reactive gliosis is the initial response and ignites detrimental pathways, then non-specific NSAID administration that targets COX-1 may only be advantageous at onset to minimize microglial activation as COX inhibition later in the cycle may curtail inflammatory-resolving mediators. Alternatively, if the primary insult is neuronal stress, then COX-2 specific inhibitors may be more appropriate early in the process to prevent excitotoxicity followed by COX-1 inhibition to suppress secondary microgliosis. 

In contrast to globally defining neuroinflammation as "bad", it is necessary to evaluate the benefit of glial activation and to consider that neurodegenerative disease progresses due to a deficient neuroimmune response. Age-related changes in microglia morphology, reactivity, and mitotic ability have suggested that microglia enter a "cellular senescence" that reduces the phagocytic removal of disease-related pathology, ultimately accelerating neurodegenerative disease [191,192]. Therefore, chronic inhibition of microglial activation during normal aging may preserve the microglial population during normal aging, however NSAID administration upon presence of disease-related pathology may worsen an already compromised microglial response. In addition, research is necessary to explore the anti-inflammatory and neuroprotective actions of COX-2 activity [137,193,194,195] to determine when, if at all, coxib administration would be favorable. Dissecting the integrity of the cellular players and the molecular pathways initiated by glial reactivity may provide more targeted treatments that specifically resolve the harmful aspects of neuroinflammation while retaining its innate benefit.

Finally, focused investigation and enhancement of the particular NSAIDs that impact COX-independent signaling, enhance antioxidant properties, and discourage plaque formation may hold greater potential independently or in conjunction with other therapies in combating neurodegeneration and delaying behavioral deficits, thereby improving daily life during the extended lifespan.
